# The Search for an Outcome Variable That Measures Both Quality and Processes in Cardiac Surgery: Comparing the Quality Process Index and Mortality

**DOI:** 10.3390/healthcare11101419

**Published:** 2023-05-14

**Authors:** Paulien C. Hoefsmit, Evert K. Jansen, Ronald J. M. M. Does, H. Reinier Zandbergen

**Affiliations:** 1 Department of Cardiothoracic Surgery, Amsterdam University Medical Centre, 1081HV Amsterdam, The Netherlands; 2 Department of Business Analytics, Amsterdam Business School, University of Amsterdam, 1081TV Amsterdam, The Netherlands

**Keywords:** quality improvement, process improvement, cardiac surgery, clinical data, performance indicator, data-driven, efficiency, index

## Abstract

Background: The translation of a large quantity of data into valuable insights for daily clinical practice is underexplored. A considerable amount of information is overwhelming, making it difficult to distill and assess quality and processes at the hospital level. This study contributes to this necessary translation by developing a Quality Process Index that summarizes clinical data to measure quality and processes. Methods: The Quality Process Index was constructed to enable retrospective analyses of quality and process evolution from 2011 to 2021 for various surgery types in the Amsterdam Cardiosurgical Database (n = 5497). It is presented alongside mortality rates, which are the golden standard for quality measurement. The two outcome variables are compared as quality and process measurement options. Results: Results showed that the mean Quality Process Index appeared rather stable, even though analysis of variance found that the mean Quality Process Index differed significantly over the years (*p* < 0.001). The 30-day and 120-day mortality rates appeared to fluctuate more, but interestingly, we failed to reject the null hypothesis of equal means. The Quality Process Index and mortality rates were statistically negatively correlated, and the extent of correlation was more pronounced with the 120-day mortality rate, as computed using the Pearson correlation coefficient r (30-day $r_{QPI,30}$ = −0.07, *p* < 0.001 and 120-day mortality rates $r_{QPI,120}$ = −0.12, *p* < 0.001). Conclusions: The Quality Process Index seeks to address the need to translate data for quality and process improvement in healthcare. While mortality remains the most impactful outcome measure, the Quality Process Index provides a more stable and comprehensive measurement of quality and process improvement or deterioration in healthcare. Therefore, the Quality Process Index as a quantification reinforces the understanding of the definition of quality and process improvement.

## 1. Introduction

Quality and process improvement is of paramount importance in cardiac surgery, reflected by the rich history of data collection that started with the collection and analysis of outcome and performance data [1,2]. Accompanied by the establishment of large regional and national databases, quality and process improvement initiatives have subsequently developed extensive reports on mortality and complication rates. Common purposes of these databases are to provide benchmarks for best practices, identify variations in care processes, stimulate quality improvement initiatives, and provide evidence-based guidelines. The first database was initiated by the United States Department of Veterans Affairs in 1971 [3]. In 1990 in the US, the Society of Thoracic Surgeons developed an adult cardiac surgery database to improve quality and patient safety by monitoring quality and performance measures [4]. On the European level, collaborative registries for benchmarking and improving peri-operative care are all included in the European Association of Cardiothoracic Surgery database for adult cardiac surgery, which contains information on approximately 100,000 surgical interventions [5]. In 2007, the Netherlands Association for Cardiothoracic Surgery initiated the Adult Cardiac Surgery Database to monitor the quality of care by analyzing mortality and postoperative outcomes [6]. The intention is to provide transparency to patients, doctors, external supervisors, and nationwide insurance companies. The Amsterdam Cardiosurgical Database was developed by surgeons to monitor the quality and processes of their cardiosurgical department. All data collection, analysis, and monitoring were performed by surgeons in the cardiosurgical department of Amsterdam. This differs from other registries, because departments often supply extracted data from hospital information systems, and data analysis is performed by others. The sense of ownership and responsibility is, therefore, more distanced when surgeons work with their own data on a daily basis to improve their clinical practice [7]. The included data directly reflected the cardiosurgical care pathway. It is well established from a variety of studies that data collection and analysis in cardiac surgery led to improved operative mortality rates [2,8,9]. Although mortality rates are the golden standard outcome measure, hospital quality, and process performance should require more informative indicators when using data for quality and process improvement in clinical practice. Shojania et al. reported that hospital mortality as an outcome-based performance measure correlates weakly with other measures of quality of care [10]. In addition, mortality rates do not encompass the entire care pathway in which much informative data are collected. It is less informative on detailed indicators, for example, accessibility (referral to treatment times), efficiency (surgical volumes, resource, and capacity use), and other quality indicators (e.g., blood loss, length of stay, complications) [11]. Shojana et al. discussed that a more robust measurement of performance data requires a combination of indicators by perhaps a score application that combines mortality with performance indicators [10]. This approach would capture a more comprehensive measurement when improving quality and performance.

In the industry, it is quite common to compare the behavior of a process or product characteristic to customer requirements (specifications) [12]. The resulting indices are called process capability indices, and relate the allowed variation determined by the specifications to the observed variation in the process. The ratios are dimensionless, large values correspond to good quality, and the complex information of a process is reduced to a single number. Many customers ask their suppliers to record capability indices of their product or process characteristics on a regular basis to ensure the right quality, measured as the percentage of non-conformities. There is a wide range of indices that cover multiple dimensions of healthcare [7,13,14,15,16,17]. In quality and process improvement methodologies, such as Lean Six Sigma, capability indices are used for diagnosing the current process and redefining the business case of the improvement project [18]. However, there has always been much confusion and misunderstanding regarding their interpretation and appropriate use [19]. Some of these concerns are also discussed in this study.

The COVID-19 pandemic highlighted the importance of quality and process improvement in healthcare delivery when dealing with scarce hospital resources and capacity. More than ever, hospitals are exposed to inefficiencies such as prolonged waiting lists, last-minute cancellations, suboptimally organized care pathways, and the use of limited capacity [20,21]. A considerable quantity of relevant data is available for quality and process improvement; however, the translation into analysis and valuable insights for daily clinical practice are underexplored. A key issue of particular concern is that a considerable quantity of information is overwhelming, such that it is difficult to distill and assess quality and performance at a hospital level. In order to provide a fair, accurate, and transparent measure of performance and outcomes, Cerfolio developed the Efficiency Quality Index (EQI) as a composite score of key metrics [7]. This index is broadly used in NYU Langone, known as one of the best-performing hospitals in the United States of America, for quality and process improvement at the hospital level. Inspired by the EQI, this study contributes to this necessary translation step by developing a performance indicator summarizing clinical data from the Amsterdam Cardiosurgical Database, which seeks to measure quality and processes: the Quality Process Index (QPI) [7]. This study addresses two main research questions:

1. How do the Quality Process Index and mortality rates compare as indicators of quality and process in healthcare in the Amsterdam Cardiosurgical Database?
2. How was the evolution of quality and processes over ten years in the Amsterdam Cardiosurgical Database?

## 2. Materials and Methods

### 2.1. Data Collection

The dataset of analysis is a subset of the Amsterdam Cardiosurgical Database from January 2011 to April 2021. Data represented the performance of the Vrije University Amsterdam Cardiac Surgery department. Since the merger of two academic centers in April, 2021, the Amsterdam Cardiosurgical Database was finalized. The dataset is comprised of the following types of surgery: (i) Coronary Artery Bypass Graft (CABG), (ii) valve (both aortic valve replacement, mitral valve repair/replacement, and tricuspid valve repair/replacement), (iii) CABG combined with a valve procedure, (iv) aortic surgery, and (v) others. Transcatheter Aortic Valve Implantation (TAVI) and Extracorporeal Membrane Oxygenation (ECMO) procedures were excluded from analysis because of the heterogeneity they would provide in the QPI. In the first place, TAVI does not require a classical sternotomy, cardiopulmonary bypass time, or postoperative admission to the ICU. Secondly, because of hospital organizational agreements, TAVI and ECMO patients were often admitted to the cardiology department and thus outside the scope of the data collection. Congenital cardiac surgeries were also omitted. Descriptive statistics included age, gender, weight, height, and Euroscore II.

### 2.2. Metrics

The metrics were chosen from the available data by cardiothoracic surgeons. Variables were defined as follows: number of procedures per year, referral to heart team discussion time (days), heart team decision to treatment time (days), X(clamp)time (minutes) and cardiopulmonary bypass time (minutes), length of stay in the intensive care unit (days), length of stay surgical ward (days), blood loss after 6 and 24 h (milliliters), mortality 30 days, mortality 120 days, re-thoracotomy, cardiac tamponade, mediastinitis and readmission (numbers). Pearson’s correlations were computed for all metrics to identify the relationship between variables (Appendix A, Table A3 Correlation matrix of metrics). It was observed that X(clamp)time and cardiopulmonary bypass time (*r* = 0.89) were highly correlated to the extent of multicollinearity, as were blood loss after 6 h and blood loss after 24 h (*r* = 0.86). To prevent counting one effect twice, only the variables deemed to capture more relevant information (cardiopulmonary bypass time and blood loss after 24 h) were included in the QPI. All metrics were combined in four phases of the care pathway (Table 1).

### 2.3. Calculation of Quality Process Index (QPI)

The QPI is a scoring system that maps the aforementioned metrics to an index, where all metrics are assigned a weight [22,23]. The weights of the metrics should represent their relative importance in assessment of quality, and were based on the surgeon’s expertise. The metrics and weights are presented in Table 1. Histograms per metric were created to identify the variation and distribution. For each metric, the mean, standard deviation (SD), and minimum and maximum values were calculated. For the variable blood loss after 24 h, the great amount of missing data (41% of datasets) was estimated by linear regression. Linear regression imputation was preferred over alternative imputation methods as it was found that regression of blood loss after 24 h on background variables was very significant (*p*-value < 0.001) and also explained a large share of the variance (adjusted R^2^ = 32.4%). If less than ten values were missing, mean substitution was performed.

It is necessary to map metrics with different dimensions and distributions into a standardized score first, before a QPI can be constructed from weighted scores. A scoring system of relative quality and processes $QPI_{i,j,k}$ as experienced by patient i for clinical metric $x_{j}$ and $x_{k}$ type of surgical procedure is proposed as follows:

(1) $$QPI_{i,j,k}=w_{j}*\frac{{\left(x_{i,j,k}-x_{worst,j,k}\right)}^{2}}{{\left(x_{best,j,k}-x_{worst,j,k}\right)}^{2}}$$

On the right-hand side, $w_{j}$ denotes a scoring weight of clinical metric $x_{j,k}$, $x_{i,j,k}$ is the value of $x_{j,k}$ observed for patient i, and $x_{worst,j,k}$ and $x_{best,j,k}$, respectively, represent the worst and best values observed for metric $x_{j}$ over all patients. For the binary variables (yes/no), the score was provided if the metric occurred (score = 0) or it did not occur (score = entire weighted score). As can easily be verified, the $QPI_{i,j,k}$ allocates the maximum score $w_{j}$ for the best value $x_{best,j,k}$ and the minimum score 0 to the worst value $x_{worst,j,k}$. All other patients receive a score between 0 and $w_{j}$. The numerator and denominator are squared, as this does not punish values closer to $x_{best,j,k}$, and increases the punishment as the observed value worsens. This quadratic is chosen since the clinical impact of some deviation in $x_{j,k}$ is generally higher near $x_{worst,j,k}$ than near $x_{best,j,k}$. In our approach, the 2nd and 98th percentile values were used as $x_{worst,j,k}$ and $x_{best,j,k}$ to address outliers, and values below the 2nd percentile and above the 98th percentile were, respectively, awarded zero and maximum points [22,23]. 

Since clinical data are relevant in context of the type of surgery, it is necessary to compute QPI as per Equation (1) separately for different surgery types. If not, the worst value amongst CABG patients would undeservedly receive a relatively good score as the value would be much better than the worst aortic surgery value observed. The quality and process scores $QPI_{i,j,k}$ for every clinical metric $x_{j,k}$ can be aggregated into a $QPI_{i}$ index for every person i by summation over all ten metrics. Note that mortality and annual number of surgeries are not a constituent metric used to construct the QPI. 

QPI, mortality, and number of procedures per year were compared over the years 2011 to 2021. The QPI and mortality rates for total and CABG are presented in figures. Given the division of the QPI per type of procedure, the decision to present only CABG in the main manuscript was made because it was the most performed type of procedure (52%). The QPI and mortality rates for the remaining four types of surgery (valves, CABG combined with valves, aortic surgery, and others) were included as Appendix A.

### 2.4. Data Analysis

Data management and analysis were performed using SPSS, version 28. To compare differences over the years, a global analysis of variance (ANOVA) F-test was used. The QPI total was compared to mortality with Pearson’s correlation. A *p*-value below 0.05 was considered statistically significant.

## 3. Results

### 3.1. Descriptive Statistics

In total, 5497 procedures were included in the analysis between 2011 and 2021. Out of those, 2860 were CABG, 1293 valves, 657 CABG and valves concomitant, 376 aortic surgery, and 311 others. In the years before the COVID-19 pandemic, the annual number of surgeries ranged from a maximum of 707 in 2012 to a minimum of 460 in 2017. In 2020, the year of the COVID-19 pandemic outbreak, 260 procedures were performed. Because of the merger of two academic centers, and the lateralization of the Vrije University Medical Centre Amsterdam cardiosurgical department in April 2021, there were only 53 procedures performed in 2021. Because of agreements during the merger, all aortic surgeries were performed at the other location in 2021, and zero aortic surgeries were recorded. When comparing the means from 2011 to 2021, only the height of the patient (*p* < 0.001) and Euroscore II (*p* = 0.003) were found to differ by analysis of variance (Table 2). The mean Euroscore II for CABG is 2.6 (SD 3.8), valve is 2.8 (SD 5.7), CABG and valves is 4.4 (SD 6.5), aortic surgery is 11.2 (SD 12.5), and others is 8.2 (SD 13.1). Euroscore II means differed significantly per type of procedure (*p* < 0.001) by analysis of variance. There was no significant difference between mortality rates after 30 days and 120 days between 2011 and 2021 (*p* = 0.783 and *p* = 0.451, respectively). Descriptive statistics can be found in Table 2.

### 3.2. Quality Process Index Total

**Figure 1:**
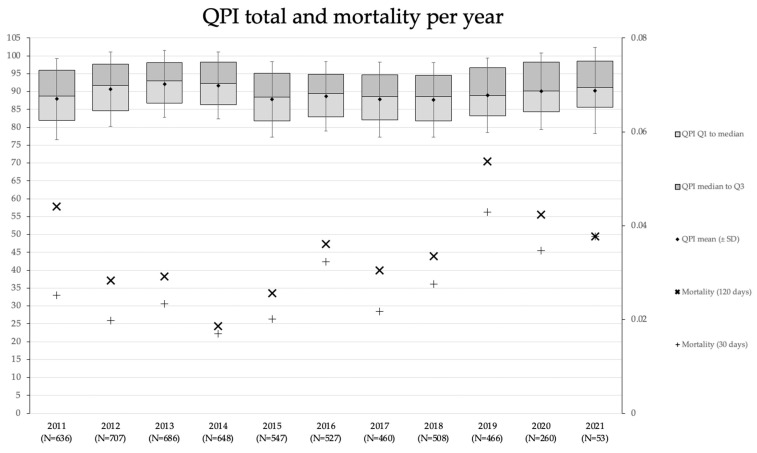
QPI total and mortality per year. QPI and mortality rates for the entire cohort over the ten-year period. The QPI distribution within a year is summarized using the first quartile (Q1), the median (Q2), the mean, and the third quartile (Q3). The standard deviation (SD) for a given year is indicated by the whiskers about the mean. The *y*-axis on the left indicates the QPI scale, whilst the y-axis on the right depicts the mortality rate scale. The number of patients for a given year is included with the *x*-axis labels.

The average QPI and mortality rates for all patients over the ten years spanned by the dataset are presented in Figure 1. The QPI ranged from a minimum of 87.7 in 2018 to a maximum of 92.1 in 2013. Although the QPI appears rather stable, it was found by analysis of variance (ANOVA) that the mean QPI differed significantly over the years (*p* < 0.001). The lack of a clearly apparent historical trend or a sudden change in QPI is to be expected as there was no standout treatment effect over this time period, such as a radical reorganization of processes. Even though a clear historical trend was absent, a decrease in the annual number of surgeries of 99 can be observed from 2014 to 2015 and in the QPI of 3.9 from 2014 to 2015. Further retrospective analysis is needed for the interpretation of this observation. It is interesting to note that we fail to reject the null hypothesis of equal means for 30-day and 120-day mortality rates over time, despite the apparent fluctuation of mortality rates over the time period. The Pearson correlation coefficient r was computed for mean QPI with mean 30-day ($r_{QPI,30}$ = −0.07, *p* < 0.001) and 120-day mortality rates ($r_{QPI,120}$ = −0.12, *p* < 0.001). There is a statistically significant negative correlation between QPI and both mortality rates, and the extent of correlation is more pronounced with the 120-day mortality rate. Recall that neither mortality rate was used to construct the QPI, such that correlation by construction is not the case. Higher QPI scores, therefore, correlate with lower mortality rates, which conforms to a priori expectations. This correlation is also apparent in Figure 1; years with low mortality rates tend to coincide with high QPI.

**Figure 2:**
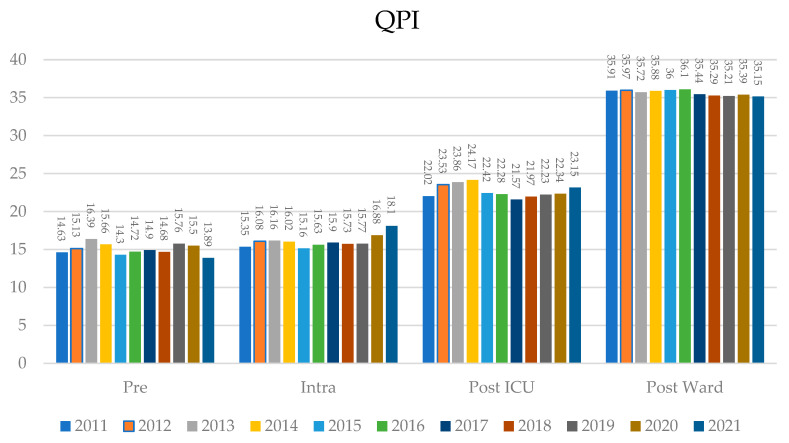
QPI. QPI per operative phase for the entire cohort over the ten-year period. The phases are divided into preoperative, intraoperative, postoperative ICU, and postoperative ward for a given year. The *y*-axis on the left indicates the QPI scale, whilst the *x*-axis depicts the operative phases. The QPI for a given year is included with the *x*-axis labels.

To identify areas for improvement per care phase, the QPI was divided into preoperative, intraoperative, postoperative intensive care, and postoperative surgical ward. Figure 2 visualizes the evolution by phase per year. Note that the weights per phase are not equally distributed, given that more metrics included in this study fall into a postoperative phase. When analyzing the QPI distribution per the four operative phases, the preoperative phase scored highest in 2013 (16.39 and SD 3.93) and lowest in 2021 (13.89 and SD 5.61). The intraoperative phase scored highest in 2021 (18.19 and SD 6.28) and lowest in 2015 (15.16 and SD 5.29). The postoperative ICU phase scored highest in 2014 (24.17 and SD 4.34) and lowest in 2017 (21.57 and SD 4.51). The postoperative ward scored highest in 2016 (36.10 and SD 3.15) and lowest in 2021 (35.15 and SD 3.61). A trend between phases could not be observed.

### 3.3. QPI CABG and Mortality Rates

**Figure 3:**
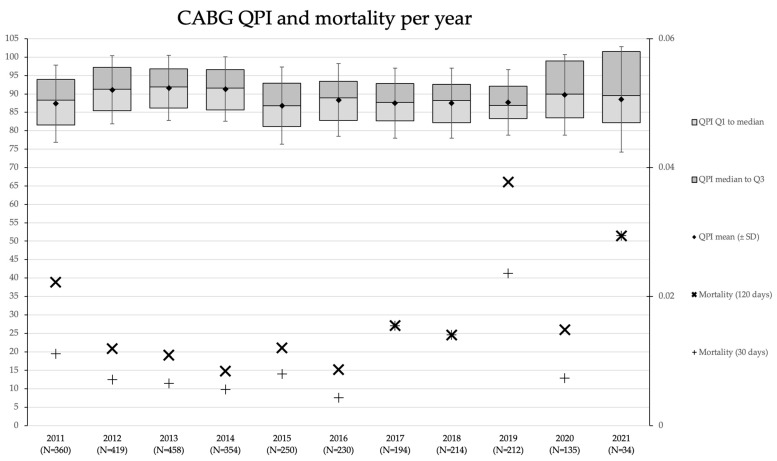
QPI CABG and mortality per year. QPI and mortality rates for the CABG over the ten-year period. The QPI distribution within a year is summarized using the first quartile (Q1), the mean (Q2), and the third quartile (Q3). The standard deviation (SD) for a given year is indicated by the whiskers about the mean. The *y*-axis on the left indicates the QPI scale, whilst the *y*-axis on the right depicts the mortality rate scale. The number of patients for a given year is included with the *x*-axis labels.

The QPI and mortality rates for CABG over ten years spanned by the dataset are presented in Figure 3. The results show that annual numbers differed until 2020 (pre-COVID) from 212 to 458 procedures. As observed for CABG as well, there is a decrease of 154 CABG surgeries from 2014 to 2015. The QPI difference between the highest and lowest is 6.0, from 96.8 and SD 11.3 (n = 368) in 2011 to 102.8 and SD 12.4 (n = 25) in 2021. The evolution of QPI for CABG closely mirrors the overall QPI, and the analysis of variance (ANOVA) once again unearths a significant difference in the mean QPI for CABG over the years (*p* < 0.001). An apparent historical trend or sudden change is also not apparent for CABG, which again can be explained by the lack of a noteworthy treatment effect. Similar to the QPI total, a decrease in QPI in 2015 is observed. In interpreting comparisons of QPI amongst different surgery types, it should be stressed that the scores are relative within a category by construction; see Equation (1). This also holds for comparing a surgery type category to the overall data. For CABG, the 30-day and 120-day mortality rates appear to fluctuate, and the highest mortality rate in the year 2019 jumps out in particular. Additionally, for the CABG subgroup, the 30-day and 120-day mortality rates were not statistically different over the years (Chi-Square statistics *p* = 0.393 and *p* = 0.227, respectively). To illustrate, the QPI in 2019 did not greatly deviate from other years (97.7 and SD 10.9).

## 4. Conclusions

Translation of a large quantity of data for quality and process improvement in daily clinical practice is underexplored. The implementation of this translation has been attempted in the literature, but a standard method has not arisen. The Quality Process Index (QPI), as presented, seeks to address this need by translating quality and process data to assess healthcare delivery. The QPI, as constructed, was specified for the performance at the cardiac surgery department at the Vrije University Medical Center in Amsterdam, the Netherlands. This study compares the QPI and mortality rates as indicators of quality and processes, and explores the evolution of quality and processes over ten years based on the Amsterdam Cardiosurgical Database. The QPI was found to differ statistically significantly over the studied time period, and the QPI exhibited a statistically significant negative correlation with mortality rates, even though we failed to establish that mortality rates differed over time. From this study, it can be concluded that although mortality remains the most impactful outcome measure, it does not reflect the general processes and quality as the QPI. As mortality fluctuated more due to small numbers, the QPI remained more stable over the observed ten years. The stability of the QPI can be explained by a historical change in clinical practice; the treatment effect of the reorganization of processes was not apparent. Since the analysis of variance of the QPI is significantly different over time, quality and process improvement or deterioration can be quantified using the data of the Amsterdam Cardiosurgical Database. It is for this reason that the QPI as quantification reinforces the understanding of the definition of quality and process improvement based on the Amsterdam Cardiosurgical Database.

## 5. Discussion

The QPI encompassed multiple quality and process indicators from the Amsterdam Cardiosurgical Database and reflected their cardiosurgical care pathway and performance. This study was based on ten years of data and included a large number of cardiac surgeries (n = 5498). The single-center Amsterdam Cardiosurgical Database was developed and monitored by surgeons themselves, which creates a high level of transparency, a strong sense of ownership, and confidence in the quality and value of the data. From its origin in 1991, the database was categorized as CABG, valves, CABG and valves, aortic surgery, and others. The group ‘others’ is quite heterogeneous and contains many different procedures, such as myxoma excisions and traumatic surgeries. In perspective, 3.5% of cardiac surgeries in the Netherlands (n = 13,750 surgeries in 16 centers) were represented by the Amsterdam Cardiosurgical Database on average in 2018 and 2019 [6]. The operative risk scored with Euroscore II was higher in this study on average in 2018 and 2019 (Netherlands Heart Registry (NHR) 1.78% versus this study 4.5%). The 30-day mortality rates in this study were higher compared to the NHR data (NHR 2.7% versus this study 3.6%). The number of CABG procedures in this study represented 3.1% of the total number of CABG procedures performed in the Netherlands on average in 2018 and 2019. The Euroscore II, according to the NHR data, was 1.42%, which was lower than the average of 2.35% in this study. Compared with the NHR data, the (raw) 30-day mortality rates in this study were 0.7% lower in 2018 (NHR 2.1% and this study 1.4%) and 1.0% higher in 2019 (NHR 1.4% and this study 2.4%) [6]. Outside the scope of this study, the significance of these differences requires further investigation.

### 5.1. How Do the Quality Process Index and Mortality Rates Compare as Indicators of Quality and Processes in the Amsterdam Cardiosurgical Database?

Inspired by ‘judge the process, not the outcome’, we hypothesized that the QPI evaluates healthcare quality and processes more robustly than mortality rates. The QPI and mortality rates were negatively correlated in this study. While mortality remains the most impactful outcome measure, it does not reflect and cannot be translated to general process and quality of healthcare delivery [7,10]. It is also important to recall the conclusions that the QPI was found to differ and exhibited a statistically significant negative correlation with mortality rates; however, mortality rates were not found to differ. This could suggest that mortality rates and the QPI as presented both capture a similar outcome effect, and also that mortality rates might not feature sufficient statistical power due to their nature of small numbers. The QPI, as constructed, may be a better method to assess overall quality and process evolution, particularly in cases where the absolute number of deaths is a very small number. Mortality rates are subject to high fluctuations due to low mortality statistics. Logistic regression analysis of the indicators and mortality rates did not yield statistically relevant results. It is noteworthy that large multi-institutional databases collect quality and performance indicators, such as readmission rates, complications, and operative mortality. For benchmarking, a combination of metrics relevant to structure, processes, and outcomes provide ratings or scores [24]. The QPI is different, given that it is developed for monitoring and improvement of quality and processes for daily clinical practice at a hospital level. 

### 5.2. How Was the Evolution of Quality and Processes over Ten Years in the Amsterdam Cardiosurgical Database?

A clear historical trend was absent in the evolution of quality and processes over the ten years in the Amsterdam Cardiosurgical Database. Nevertheless, the absence of a clear historical trend in the QPI over time does not disqualify the QPI as an indicator. First, the comparison of the QPI with mortality resulted in a negative correlation. Second, the plots over time in total and per type of surgery show that there is indeed a change that summarizes hundreds of surgeries using a consistent method. Conclusions can be drawn from the trend of quality and processes over time, as quantified by the QPI, on the cardiosurgical performance, such as the annual number of surgeries, efficiency, and, for example, the effect of COVID-19. The QPI is an overall score that summarizes the quality and processes. As observed, the year of the highest total index, 2013, did not score the highest in the intraoperative and postoperative phases. This indicates that even though the total QPI was the highest, the potential for improvement in other phases could have been possible. It does not identify individual scores per metric. If, for a particular year, the score for variable A is high but for variable B is low, and this is reversed the next year, the year scores are both similar. Therefore, the lesson from summarizing indices on performance is that the potential for quality and process improvement should also be assessed independently. Note that it is always difficult to summarize complex issues, such as surgeries and corresponding non-conformities (mortality rates), in a single number. In the manufacturing industry, these complex issues have been summarized with process capability indices for many years [19]. 

### 5.3. Reflection on Method

Indices for quality and process improvement are common in the manufacturing industry and in improvement methodologies such as Lean and Six Sigma [17,18]. In healthcare, indices are used to objectively measure and compare a variety of dimensions [13,14,15,16,17]. The difference the QPI has is that it is specifically designed for quality and processes in a hospital’s department. The QPI was calculated by the given Equitation (1) in the method section. *X_best-j,k_* and *X_worst-j,k_* represent the 2% best and 98% worst variable based on the complete dataset for each type of surgery to enable comparison over the years. The 2% cutoff was chosen to correct for extreme values. We used the squared method in the calculation to punish deviations. For example, a postoperative blood loss difference of 10 cc and 110 cc is less impactful for a patient than a similar increase from 1400 cc to 1500 cc. As an effect, values close to *X_best-j,k_* receive an almost perfect score, while scores half of *X_worst-j,k_* still receive a reasonable score (3/4 * weight). Without this correction, a number of QPI components occurred where almost everyone scored perfectly or, on the contrary, scored almost nothing. There is perhaps controversy regarding the chosen weights, which is an intrinsic disadvantage of indices. We aimed to quantify an unmeasurable value of quality and processes, so for this purpose, one may always be exposed to the disadvantage of weights. The weights were chosen by cardiac surgeons themselves, but the weights in the equitation can be adjusted for other purposes and developments if necessary. Even though obvious in clinical practice, a correlation matrix of the indicators showed that most indicators were weakly correlated (Appendix A: Table A3). To a certain extent, this indicates that diversity in dimensions is measured by the QPI. Even though a small correlation exists, this can be explained by rationality. For instance, the amount of postoperative blood loss is associated with a rethoracotomy. Nonetheless, the chosen indicators were considered relevant for quality and processes since, for instance, the amount of blood loss is related to quality and operative factors, while the number of re-thoracotomies is informative for operating room efficiency as well.

### 5.4. In Perspective: QPI versus EQI

The QPI was inspired by the EQI, which is a scoring system of the overall index for efficiency and quality at a procedure or departmental level in hospitals [7]. The QPI differs from the EQI because the formula is quadratic, as explained in the method section. The EQI was developed for clinical practice instead of research purposes. Even though the QPI is focused on overall cardiac surgery performance, the EQI is procedure dependent and is perhaps, therefore, more specific. This score was used to identify which surgeons could improve in a positive learning culture by learning from each other. The purpose of measuring performance for improvement on a surgeon level is also suggested by the designed index of Cerfolio, implemented in New York Langone healthcare [7].

### 5.5. Generalizability, Limitations, and Recommendations

In regard to the generalizability of the QPI approach, calculation and implementation are generalizable because of its applicability in different healthcare settings. Even though the QPI as constructed in this study was based on the Amsterdam Cardiosurgical Database, every hospital department is able to select relevant metrics, assign weights and summarize them as indices. Data collection can be performed simultaneously using large registries and databases. An index with quality and process metrics can be used to monitor and analyze deviations in healthcare delivery. Indices should be analyzed continuously and during staff meetings to accurately act on variations that negatively impact healthcare quality and processes. The limitations of this study include its retrospective design, which was based on a single-center database. Even though there are many advantages experienced by data collection and evaluation by surgeons themselves, as in the Amsterdam Cardiosurgical Database and recommended by the EQI, there are still limitations that should be further explored in regard to the quality of the data, such as how to address inconsistencies and amendments. We explored the approach of summarizing indices to quantify quality and processes, which contributed to the meaning of quality and process ‘improvement’ or ‘deterioration’ [25]. Even though the QPI has inherent limitations of being a single metric constructed from empirically chosen weights, it provides useful insights into long-term trends. The QPI approach is a foundation that can be further developed. In the future, it would be interesting to explore the differences in QPI using procedure-specific metrics. For example, the type and size of valves for valve surgery or the number of anastomoses for CABG. Nevertheless, this study was limited to the available data. However, relevant metrics not available in this study were quality of life assessment, number of repeated heart team discussions, number of last-minute cancellations, and operating room utilization. Based on this study, the QPI as a summarizing indicator can be further developed to test the expected performance based on risk scores as Euroscore II. A substantial number of potential opportunities exist for data-driven quality and process improvement in daily clinical practice, such as (un)supervised learning and the use of machine learning techniques [26]. This, however, together with the QPI, should be further explored to identify which approach would ultimately be best for improving daily clinical care, specifically cardiac surgery.

In conclusion, the QPI contributes to the quantification and meaning of quality and process improvement based on the Amsterdam Cardiosurgical Database. Important lessons learned are the use of reliable data that create a sense of urgency and ownership by stakeholders, timely evaluations of data for translation to clinical practice, and opportunities to use data to improve daily clinical practice and quality and processes of care.

## Figures and Tables

**Table 1:** Metrics and weight per metric for QPI.

Phase	Metric	$\mathrm{Weight}\;(w_{j})$
Preoperative	Referral to decision time (days)	10
	Referral to treatment time (days)	10
Intraoperative	Cardiopulmonary bypass time (minutes)	15
	Rethoracotomy (binary: yes/no)	10
Postoperative ICU	Length of stay in intensive care unit (days)	15
	Blood loss after 24 h (milliliters)	15
Postoperative ward	Length of stay in surgical ward (days)	15
	Cardiac tamponade (binary: yes/no)	10
	Mediastinitis (binary: yes/no)	10
	Readmissions (binary: yes/no)	10
	Total (maximum)	120

**Table 2:** Descriptive statistics per year (means).

	Procedures	Age (Years)	Weight (kg)	Gender	Height (cm)	Euroscore II	Mortality 30 Days	Mortality 120 Days
2011	636	67.70 (SD 10.38)	82.52 (SD 13.855)	0.24 (SD 0.43)	173.42 (SD 8.89)	3.108 (SD 4.5839)	0.025	0.044
2012	707	67.26 (SD 10.17)	82.88 (SD 14.66)	0.26 (SD 0.44)	173.74 (SD 9.60)	3.51 (SD 5.72)	0.024	0.028
2013	686	67.94 (SD 10.0)	82.59 (SD 15.52)	0.24 (SD 0.43)	173.50 (SD 8.74)	3.50 (SD 6.65)	0.023	0.029
2014	648	66.76 (SD 10.75)	83.48 (15.24)	0.24 (SD 0.43)	173.97 (SD 9.60)	3.50 (SD 6.57)	0.017	0.019
2015	547	67.82 (SD 10.20)	83.24 (SD 15.51)	0.26 (SD 0.44)	173.23 (SD 9.46)	3.48 (SD 6.03)	0.020	0.026
2016	527	67.99 (SD 9.59)	82.92 (SD 15.80)	0.29 (SD 0.45)	173.18 (SD 9.82)	3.56 (SD 6.90)	0.032	0.036
2017	460	67.34 (SD 10.67)	83.84 (SD 15.31)	0.27 (SD 0.45)	174.94 (SD 9.06)	3.93 (SD 7.66)	0.022	0.030
2018	508	67.70 (SD 10.31)	82.16 (15.04)	.25 (SD 0.436)	174.92 (9.05)	4.22 (SD 8.69)	0.028	0.033
2019	466	66.81 (SD 10.53)	82.20 (SD 16.27)	0.29 (SD 0.45)	174.39 (SD 9.82)	4.71 (SD 9.39)	0.043	0.054
2020	260	66.12 (SD 11.08)	85.28 (SD 16.26)	0.24 (SD 0.43)	175.56 (9.95)	3.01 (SD 6.13)	0.035	0.042
2021	53	68.46 (SD 10.42)	85.23 (SD 15.15)	0.17 (SD 0.379)	175.83 (SD 9.05)	1.76 (SD 1.67)	0.038	0.038
Total	5498	67.4 (10.33)	83.01 (SD 15.26)	0.26 (SD 0.44)	173.96 (SD 9.38)	3.62 (SD 6.84)		

One-way ANOVA test for comparing means per year: age (*p* = 0.167), weight (*p* = 0.219), gender (*p* = 0.351), height (*p* ≤ 0.001), and Euroscore II (*p* = 0.003). Chi-square statistics mortality 30 days per year: *p* = 0.783. Chi-Square statistics mortality 120 days per year: *p* = 0.451.

**Figure A1:**
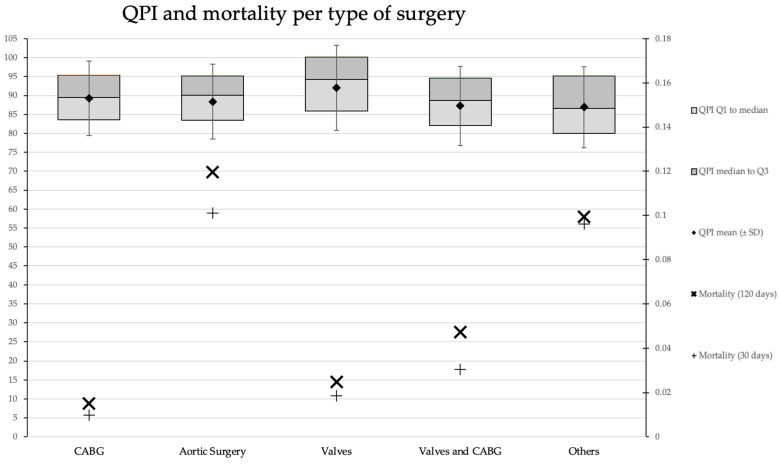
QPI total and mortality per type of surgery. QPI and mortality rates for the entire cohort per type of surgery. The QPI distribution per type of surgery is summarized using the first quartile (Q1), the median (Q2), the mean, and the third quartile (Q3). The standard deviation (SD) for a given surgery type is indicated by the whiskers about the mean. The *y*-axis on the left indicates the QPI scale, whilst the *y*-axis on the right depicts the mortality rate scale.

**Figure A2:**
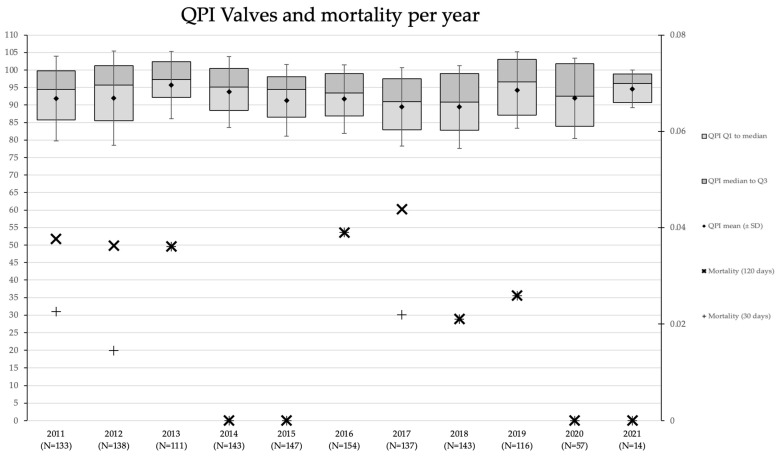
QPI valves and mortality per year. QPI and mortality rates for the type of surgery valves over the ten-year period. The QPI distribution within a year is summarized using the first quartile (Q1), the median (Q2), the mean, and the third quartile (Q3). The standard deviation (SD) for a given year is indicated by the whiskers about the mean. The *y*-axis on the left indicates the QPI scale, whilst the *y*-axis on the right depicts the mortality rate scale. The number of patients for a given year is included with the *x*-axis labels.

**Figure A3:**
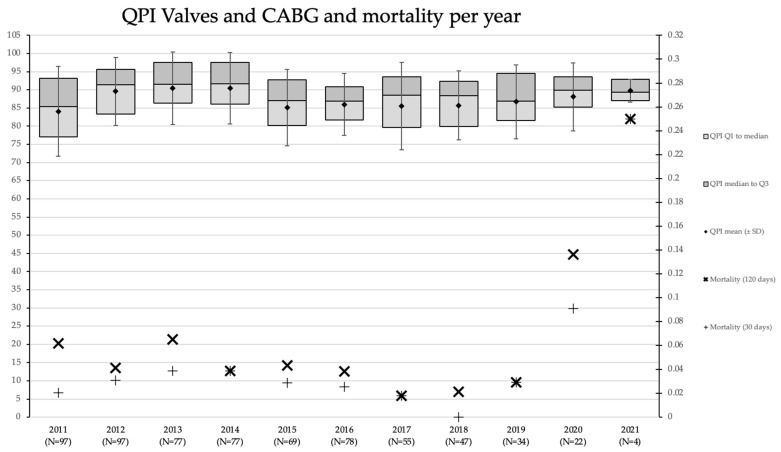
QPI Valves and CABG and mortality per year. QPI and mortality rates for the type of surgery valves and CABG over the ten-year period. The QPI distribution within a year is summarized using the first quartile (Q1), the median (Q2), the mean, and the third quartile (Q3). The standard deviation (SD) for a given year is indicated by the whiskers about the mean. The *y*-axis on the left indicates the QPI scale, whilst the *y*-axis on the right depicts the mortality rate scale. The number of patients for a given year is included with the *x*-axis labels.

**Figure A4:**
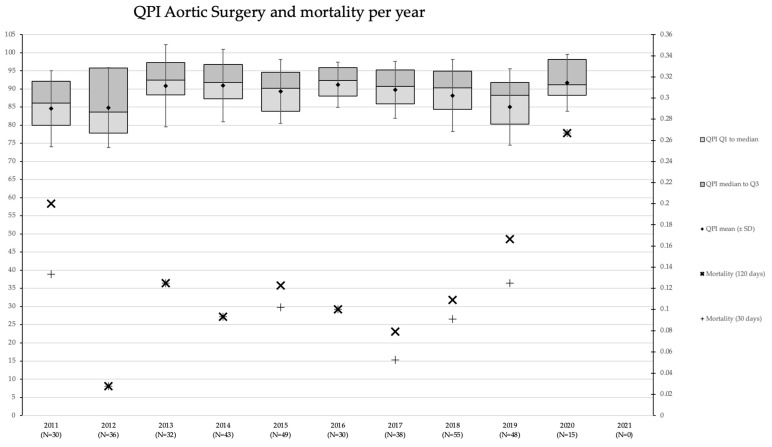
QPI Aortic surgery and mortality per year. QPI and mortality rates for the type of surgery aortic surgery over the ten-year period. The QPI distribution within a year is summarized using the first quartile (Q1), the median (Q2), the mean, and the third quartile (Q3). The standard deviation (SD) for a given year is indicated by the whiskers about the mean. The *y*-axis on the left indicates the QPI scale, whilst the *y*-axis on the right depicts the mortality rate scale. The number of patients for a given year is included with the *x*-axis labels.

**Figure A5:**
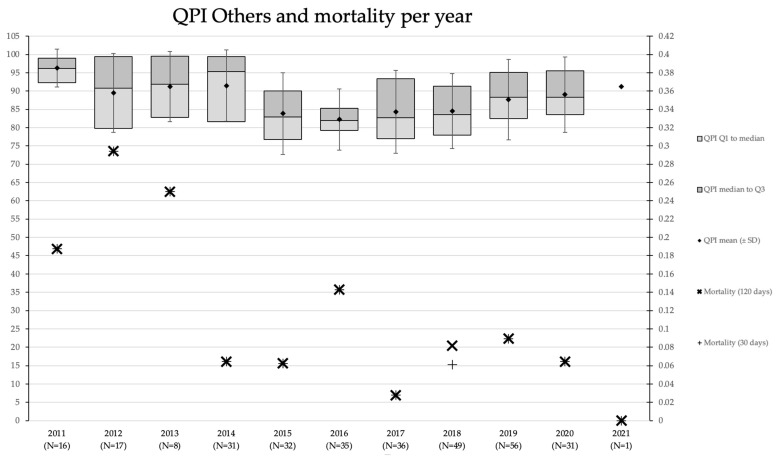
QPI Others and mortality per year. QPI and mortality rates for the type of surgery others over the ten-year period. The QPI distribution within a year is summarized using the first quartile (Q1), the median (Q2), the mean, and the third quartile (Q3). The standard deviation (SD) for a given year is indicated by the whiskers about the mean. The *y*-axis on the left indicates the QPI scale, whilst the *y*-axis on the right depicts the mortality rate scale. The number of patients for a given year is included with the *x*-axis labels.

## Data Availability

Data are unavailable due to privacy restrictions.

## Appendix A. Quality Process Index per Type of Surgery

Content

- Table A1: QPI total and per phase per year (means).
- Table A2: Descriptive characteristics total and per type of procedure (means).
- Table A3: Correlation matrix of metrics.
- Figure A1: QPI total and mortality per type of surgery.
- Figure A2: QPI valves and mortality per year.
- Figure A3: QPI Valves and CABG and mortality per year.
- Figure A4: QPI Aortic surgery and mortality per year.
- Figure A5: QPI Others and mortality per year.

Table A1 and Table A2 present the mean QPI in total and per phase and the descriptive characteristics in total and per type of procedure. Table A1 supports Figure 2 QPI per operative phase in the original file. Table A3 presents the correlation matrix of metrics included in the Amsterdam Cardiosurgical Database.

Analyzing the index and mortality per year for a specific type of surgery enables us to identify the evolution more specifically. More detailed information can be found in Appendix A.

Figure A1 presents the QPI per type of surgery for the entire study period. The QPI presented that the index was highest, and mortality rates were lowest for valves and CABG. The mortality rates were highest for aortic surgery, even though the QPI did not vary considerably compared with respect to the types of procedures. The QPI for CABG was 89.29 (SD 9.80), for aortic surgery was 88.40 (SD 9.89), for valves was 92.05 (SD 11.19), for CABG and valves was 87.28 (SD 10.49), and for others was 86.92 (SD 10.67).

Figure A2, Figure A3, Figure A4 and Figure A5 visualize the QPI per various types of surgeries and are presented alongside mortality rates. For all types of surgeries, QPI per various types is rather stable, and mortality rates appear to fluctuate more. This observation is similar to the QPI total and QPI CABG.

**Table A1:** Overall EQI and EQI per phase per year (means).

	Total	Pre	Intra	Post ic	Post Ward
2011	87.91 (SD 11.37)	14.63 (SD 4.71)	15.35 (SD 5.36)	22.02 (SD 5.71)	35.91 (SD 2.96)
2012	90.70 (SD 10.44)	15.13 (SD 4.55)	16.08 (SD 5.28)	23.53 (SD 5.16)	35.97 (SD 2.78)
2013	92.12 (SD 9.36)	16.39 (SD 3.93)	16.16 (SD 5.12)	23.86 (SD 5.03)	35.72 (SD 2.89)
2014	91.73 (SD 9.36)	15.66 (SD 4.26)	16.02 (SD 4.98)	24.17 (SD 4.34)	35.88 (SD 2.89)
2015	87.88 (SD 10.57)	14.30 (SD 4.68)	15.16 (SD 5.29)	22.42 (SD 5.46)	36.00 (SD 3.16)
2016	88.73 (SD 9.72)	14,72 (SD 4.60)	15.63 (SD 4.94)	22.28 (SD 4.48)	36.10 (SD 3.15)
2017	87.77 (SD 10.48)	14.90 (SD 4.72)	15.90 (SD 5.69)	21.57 (SD 4.51)	35.44 (SD 3.94)
2018	87.67 (SD 10.39)	14.68 (SD 4.76)	15.73 (SD 5.68)	21.97 (SD 4.24)	35.29 (SD 3.36)
2019	88.98 (SD 10.42)	15.76 (SD 3.97)	15.77 (SD 5.92)	22.23 (SD 3.95)	35.21 (SD 3.63)
2020	90.10 (SD 10.72)	15.50 (SD 4.24)	16.88 (SD 6.14)	22.34 (SD 3.86)	35.39 (SD 3.20)
2021	90.30 (SD 12.04)	13.89 (SD 5.61)	18.10 (SD 6.28)	23.15 (SD 3.20)	35.15 (SD 3.61)
Total	89.51 (SD 10.40)	15.17 (SD 4.51)	15.85 (SD 5.40)	22.76 (SD 4.87)	35.73 (SD 3.18)

ANOVA test difference between EQI total and groups *p* < 0.001.

**Table A2:** Descriptive characteristics total and per type of procedure (means).

	Procedures	Age (Years)	Weight (kg)	Height (cm)	Gender	Euroscore II
Total	5498	67.4 (SD 10.3)	83.0 (SD 15.3)	174.0 (SD 9.4)	Male 4083 Female 1415	3.6 (SD 6.8)
CABG	2860	67.3 (SD 9.33)	84.2 (SD 14.7)	174.6 (SD 8.9)	Male 2386 Female 474	2.6 (SD 3.8)
Valve	1293	68.3 (SD 10.6)	81.2 (SD 15.5)	172.7 (SD 9.9)	Male 782 Female 511	2.8 (SD 5.7)
Aortic surgery	376	63.1 (SD 11.2)	84.2 (SD 16.3)	176.9 (SD 10.3)	Male 261 Female 115	11.2 (SD 12.5)
CABG + valve	657	68.4 (SD 10.6)	81.8 (SD 14.5)	172.2 (SD 8.9)	Male 473 Female 184	4.4 (SD 6.5)
Others	311	60.5 (SD 14.7)	81.1 (SD 18.4)	173.7 (SD 10)	Male 181 Female 131	8.2 (SD 13.1)

Compare mean ANOVA per year: age *p* < 0.001, weight *p* < 0.001, gender *p* < 0.001, height *p* < 0.001, and Euroscore II *p* < 0.001.

**Table A3:** Correlation matrix of metrics.

Correlations															
		Readmissions	Referral to Treatment Times (Days)	Length of Stay in Intensive Care Unit (Days)	Cardiopulmonary Bypass Time (Minutes)	X(clamp)time	Bloodloss 24 h	Bloodloss 6 h	Re-thoracotomy	Cardiac tamponade	Mediastinitis	Referral to Decision Time (Days)	Length of Stay in Surgical Ward	Mortality 30 Days	Mortality 120 Days
Readmissions	Pearson Correlation	1	−0.064 **	0.052 **	−0.049 **	−0.061 **	0.018	0.024	−0.016	−0.013	−0.005	−0.017	0.069 **	0.018	0.013
	Sig. (2-tailed)		0.000	0.000	0.000	0.000	0.300	0.164	0.228	0.352	0.719	0.197	0.000	0.182	0.351
	N	5498	5493	5438	5480	5480	3244	3244	5498	5498	5498	5493	5398	5497	5497
Referral to treatment times (days)	Pearson Correlation	−0.064 **	1	−0.049 **	0.117 **	0.143 **	−0.038 *	−0.044 *	0.000	0.016	0.023	0.129 **	−0.017	−0.074 **	−0.081 **
	Sig. (2-tailed)	0.000		0.000	0.000	0.000	0.029	0.012	0.977	0.241	0.088	0.000	0.213	0.000	0.000
	N	5493	5493	5434	5476	5476	3244	3244	5493	5493	5493	5493	5394	5493	5493
Length of stay in intensive care unit (days)	Pearson Correlation	0.052 **	−0.049 **	1	0.147 **	0.079 **	0.154 **	0.136 **	0.114 **	0.099 **	0.005	−0.008	0.201 **	0.109 **	0.250 **
	Sig. (2-tailed)	0.000	0.000		0.000	0.000	0.000	0.000	0.000	0.000	0.705	0.533	0.000	0.000	0.000
	N	5438	5434	5438	5427	5426	3243	3243	5438	5438	5438	5434	5398	5438	5438
Cardiopulmonary bypass time (minutes)	Pearson Correlation	−0.049 **	0.117 **	0.147 **	1	0.893 **	0.214 **	0.170 **	0.078 **	0.082 **	0.002	0.021	0.203 **	0.138 **	0.135 **
	Sig. (2-tailed)	0.000	0.000	0.000		0.000	0.000	0.000	0.000	0.000	0.886	0.125	0.000	0.000	0.000
	N	5480	5476	5427	5480	5479	3244	3244	5480	5480	5480	5476	5388	5480	5480
X(clamp)time	Pearson Correlation	−0.061 **	0.143 **	0.079 **	0.893 **	1	0.211 **	0.174 **	0.074 **	0.089 **	0.011	0.044 **	0.183 **	0.074 **	0.077 **
	Sig. (2-tailed)	0.000	0.000	0.000	0.000		0.000	0.000	0.000	0.000	0.430	0.001	0.000	0.000	0.000
	N	5480	5476	5426	5479	5480	3244	3244	5480	5480	5480	5476	5387	5480	5480
Bloodloss 24 h	Pearson Correlation	0.018	−0.038 *	0.154 **	0.214 **	0.211 **	1	0.864 **	0.372 **	0.100 **	−0.001	0.033	0.139 **	0.065 **	0.069 **
	Sig. (2-tailed)	0.300	0.029	0.000	0.000	0.000		0.000	0.000	0.000	0.950	0.062	0.000	0.000	0.000
	N	3244	3244	3243	3244	3244	3244	3239	3244	3244	3244	3244	3229	3244	3244
Bloodloss 6 h	Pearson Correlation	0.024	−0.044 *	0.136 **	0.170 **	0.174 **	0.864 **	1	0.360 **	0.074 **	0.002	0.054 **	0.097 **	0.057 **	0.061 **
	Sig. (2-tailed)	0.164	0.012	0.000	0.000	0.000	0.000		0.000	0.000	0.912	0.002	0.000	0.001	0.000
	N	3244	3244	3243	3244	3244	3239	3244	3244	3244	3244	3244	3229	3244	3244
Re-thoracotomy	Pearson Correlation	−0.016	0.000	0.114 **	0.078 **	0.074 **	0.372 **	0.360 **	1	0.323 **	0.142 **	0.019	0.110 **	0.085 **	0.101 **
	Sig. (2-tailed)	0.228	0.977	0.000	0.000	0.000	0.000	0.000		0.000	0.000	0.153	0.000	0.000	0.000
	N	5498	5493	5438	5480	5480	3244	3244	5498	5498	5498	5493	5398	5497	5497
Cardiac tamponade	Pearson Correlation	−0.013	0.016	0.099 **	0.082 **	0.089 **	0.100 **	0.074 **	0.323 **	1	0.062 **	0.017	0.124 **	0.046 **	0.072 **
	Sig. (2-tailed)	0.352	0.241	0.000	0.000	0.000	0.000	0.000	0.000		0.000	0.211	0.000	0.001	0.000
	N	5498	5493	5438	5480	5480	3244	3244	5498	5498	5498	5493	5398	5497	5497
Mediastinitis	Pearson Correlation	−0.005	0.023	0.005	0.002	0.011	−0.001	0.002	0.142 **	0.062 **	1	0.011	0.066 **	0.045 **	0.069 **
	Sig. (2-tailed)	0.719	0.088	0.705	0.886	0.430	0.950	0.912	0.000	0.000		0.407	0.000	0.001	0.000
	N	5498	5493	5438	5480	5480	3244	3244	5498	5498	5498	5493	5398	5497	5497
Referral to decision time (days)	Pearson Correlation	−0.017	0.129 **	−0.008	0.021	0.044 **	0.033	0.054 **	0.019	0.017	0.011	1	0.020	−0.004	0.008
	Sig. (2-tailed)	0.197	0.000	0.533	0.125	0.001	0.062	0.002	0.153	0.211	0.407		0.139	0.753	0.547
	N	5493	5493	5434	5476	5476	3244	3244	5493	5493	5493	5493	5394	5493	5493
Length of stay in surgical ward (days)	Pearson Correlation	0.069 **	−0.017	0.201 **	0.203 **	0.183 **	0.139 **	0.097 **	0.110 **	0.124 **	0.066 **	0.020	1	−0.059 **	0.002
	Sig. (2-tailed)	0.000	0.213	0.000	0.000	0.000	0.000	0.000	0.000	0.000	0.000	0.139		0.000	0.895
	N	5398	5394	5398	5388	5387	3229	3229	5398	5398	5398	5394	5398	5398	5398
Mortality 30 days	Pearson Correlation	0.018	−0.074 **	0.109 **	0.138 **	0.074 **	0.065 **	0.057 **	0.085 **	0.046 **	0.045 **	−0.004	−0.059 **	1	0.874 **
	Sig. (2-tailed)	0.182	0.000	0.000	0.000	0.000	0.000	0.001	0.000	0.001	0.001	0.753	0.000		0.000
	N	5497	5493	5438	5480	5480	3244	3244	5497	5497	5497	5493	5398	5497	5497
Mortality 120 days	Pearson Correlation	0.013	−0.081 **	0.250 **	0.135 **	0.077 **	0.069 **	0.061 **	0.101 **	0.072 **	0.069 **	0.008	0.002	0.874 **	1
	Sig. (2-tailed)	0.351	0.000	0.000	0.000	0.000	0.000	0.000	0.000	0.000	0.000	0.547	0.895	0.000	
	N	5497	5493	5438	5480	5480	3244	3244	5497	5497	5497	5493	5398	5497	5497

** Correlation is significant at the 0.01 level (2-tailed). * Correlation is significant at the 0.05 level (2-tailed).
